# The role of lncRNA NEAT1 in human cancer chemoresistance

**DOI:** 10.1186/s12935-024-03426-x

**Published:** 2024-07-05

**Authors:** Feng Long, Xue Li, Jingyu Pan, Hailin Ye, Cuixia Di, Yong Huang, Jiawei Li, Xuan Zhou, Huiyi Yi, Qiaozhen Huang, Jing Si

**Affiliations:** 1 School of Basic Medicine, Gansu University of Chinese Medicine, Lanzhou, China; 2 Department of Medical Physics, Institute of Modern Physics, Chinese Academy of Sciences, Lanzhou, China

**Keywords:** Cancer, Chemotherapy resistance, NEAT1, Mechanism of action

## Abstract

Chemotherapy is currently one of the most effective methods in clinical cancer treatment. However, chemotherapy resistance is an important reason for poor chemotherapy efficacy and prognosis, which has become an urgent problem to be solved in the field of cancer chemotherapy. Therefore, it is very important to deeply study and analyze the mechanism of cancer chemotherapy resistance and its regulatory factors. Long non-coding RNA nuclear paraspeckle assembly transcript 1 (LncRNA NEAT1) has been shown to be closely associated with chemotherapy resistance in cancer. NEAT1 induces cancer cell resistance to chemotherapeutic drugs by regulating cell apoptosis, cell cycle, drug transport and metabolism, DNA damage repair, EMT, autophagy, cancer stem cell characteristics, and metabolic reprogramming. This indicates that NEAT1 may be an important target to overcome chemotherapy resistance and is expected to be a potential biomarker to predict the effect of chemotherapy. This article summarizes the expression characteristics and clinical characteristics of NEAT1 in different cancers, and deeply discusses the regulatory role of NEAT1 in cancer chemotherapy resistance and related molecular mechanisms, aiming to clarify NEAT1 as a new target to overcome cancer chemotherapy resistance and the feasibility of chemotherapy sensitizers, with a view to providing a potential therapeutic direction for overcoming the dilemma of cancer resistance in the future.

## Introduction

Cancer, as the second leading cause of death in the world, is a serious threat to human health and has become a major public health problem worldwide [1]. The treatment of cancer mainly includes surgical treatment, endocrine therapy, targeted therapy, immunotherapy, radiotherapy and chemotherapy [2]. Chemotherapy is one of the most effective means of clinical treatment of cancer at present. It can significantly reduce the risk of long-term recurrence of cancer and improve the overall survival and quality of life of patients. It has become the main treatment method for patients with systemic cancer, patients with advanced cancer without surgical indications and patients with recurrent and metastatic cancer [3–5]. However, with the continuous use of chemotherapy drugs, some patients will develop resistance to chemotherapy drugs, resulting in poor chemotherapy effect and poor prognosis, which has become a very difficult scientific problem in the field of cancer chemotherapy. Clinical data show that more than 70% of ovarian cancer patients relapse after receiving continuous chemotherapy for 12–18 months due to drug resistance to chemotherapeutic drugs, and the 5-year survival rate is less than 50% [6]. Therefore, it is undoubtedly an effective measure to solve the problem of clinical cancer chemotherapy resistance and poor prognosis by clarifying the molecular mechanism of driving cancer to produce chemotherapy resistance, deeply analyzing and finding potential new targets for cancer chemotherapy sensitization.

**Fig. 1:**
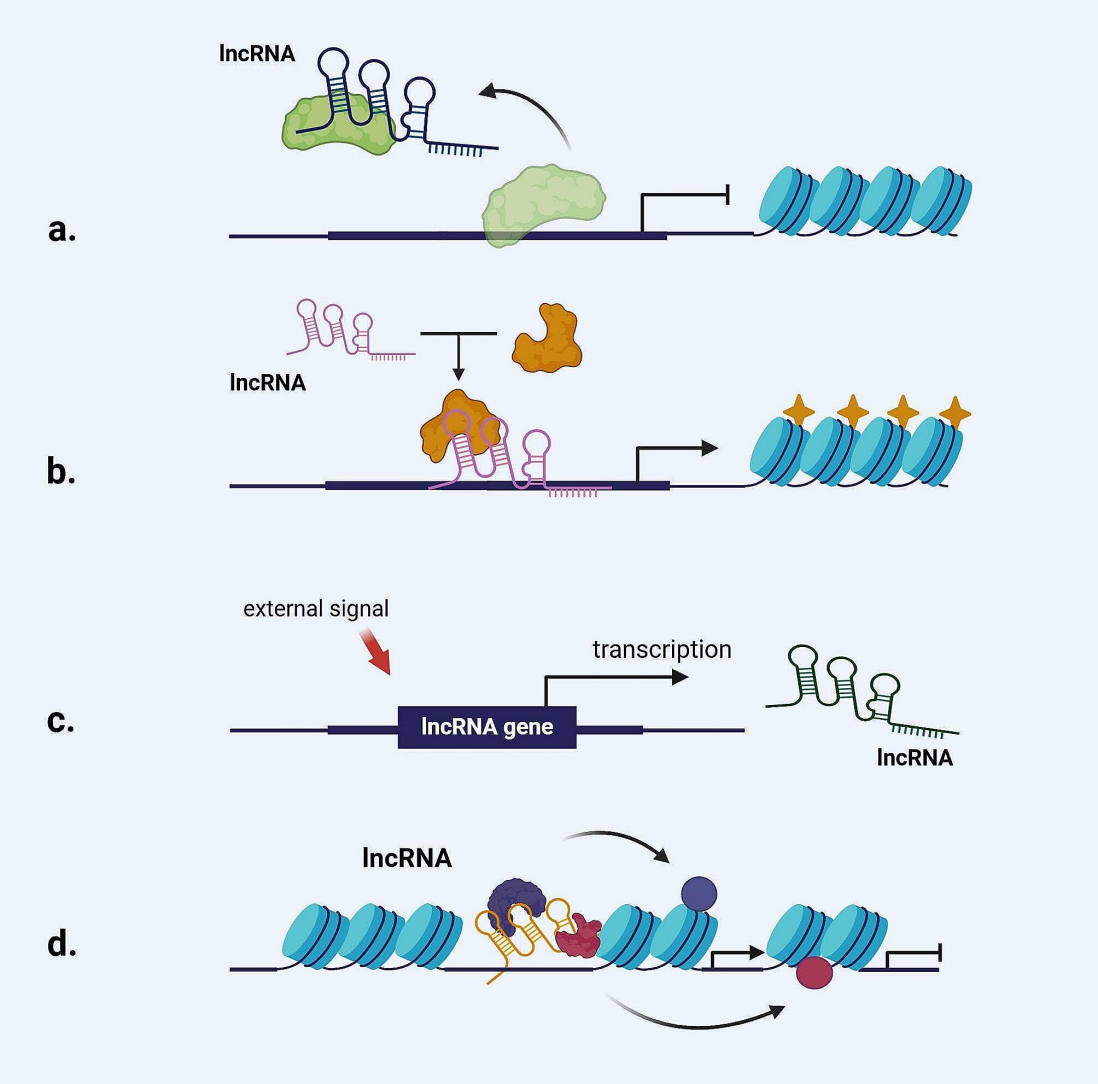
The function of lncRNA. (**a**) decoy molecule. (**b**) guide molecule. (**c**) signal molecule. (**d**) scaffold molecule

Chemotherapy induced drug resistance can be regarded as an adaptive change of cancer cells to chemotherapeutic drugs [7]. Cancer cells are resistant to chemotherapeutic drugs by altering cell cycle checkpoints, drug transport and metabolism, DNA damage repair, apoptosis, EMT, autophagy, cancer stem cell properties, and metabolic reprogramming [8–10]. Therefore, exploring the important regulatory factors that regulate the biological behavior of cancer cells to produce drug resistance and their mechanism of action is an urgent need to find potential new targets for chemotherapy sensitization and solve clinical cancer chemotherapy resistance. Recent studies have shown that long non-coding RNA (LncRNA) can affect the chemotherapy resistance of cancer by inhibiting apoptosis, promoting DNA damage repair and changing drug metabolism, and plays an important regulatory role in cancer chemotherapy resistance [11, 12]. LncRNAs are a class of non-coding RNA molecules with a length of more than 200 nucleotides that are transcribed from different regions of the genome by RNA polymerase II. They are widely present in cells and play an important role in various biological processes such as development, metabolism, immunity, and cancer [13]. Although LncRNA does not encode proteins, it can affect gene expression through chromatin remodeling, transcriptional regulation, splicing regulation, translation regulation, miRNA sponge and other mechanisms [14]. According to their different structures and characteristics, lncRNAs can be divided into: intron lncRNA, bidirectional lncRNA, intergenic lncRNA, enhancer lncRNA, sense lncRNA, antisense lncRNA, promoter lncRNA, and small nucleolar RNA terminal lncRNA [15–20]. LncRNAs typically have complex secondary structures, such as single-stranded, double-stranded, and circular. They act as decoy molecule, guide molecule, signal molecule, and scaffold molecule to interact with DNA, RNA, and proteins, respectively, thereby regulating the occurrence and development of cancer [20](Fig. 1). More and more evidence suggests that lncRNA plays a complex and multifaceted role in chemotherapy resistance. It can affect chemotherapy resistance by inhibiting apoptosis, promoting cancer stem cell characteristics, affecting cell metabolism, inducing autophagy, affecting cell cycle regulation, reshaping DNA repair capacity, and changing drug metabolism and transport [21]. Jiang et al. [22]. found that the lncRNA DDIT4-AS1 could promote paclitaxel resistance in triple-negative breast cancer by recruiting the RNA-binding protein AUF1 to stabilize DDIT4 mRNA and enhance the interaction between DDIT4 and AUF1, thereby inducing autophagy. MEIS1 is a member of the TALE family and enhances cancer sensitivity to oxaliplatin by preventing DNA damage repair. LncRNA ELFN1-AS1 promotes DNA methylation and H3K27me3 in the promoter region of MEIS1 by guiding EZH2-DNMT3 a to locate in the promoter region of MEIS1, inhibits the transcription of MEIS1, and thus promotes oxaliplatin resistance in colorectal cancer by preventing DNA damage repair [23]. Long non-coding RNA nuclear paraspeckle assembly transcript 1(LncRNA NEAT1) is a highly conserved intergenic LncRNA, which was first discovered in mammalian genome-wide screening [24]. Studies have shown that NEAT1 is associated with the development of a variety of human diseases, including non-cancerous lesions such as sepsis and neurodegenerative lesions, as well as a variety of cancers such as hepatocellular carcinoma [25–27]. NEAT1 has different expression patterns in different cancers and can affect the occurrence, development and metastasis of many cancers by acting as oncogenes or oncogenes, and is a potential diagnostic and therapeutic target for cancer [24]. Recent studies have shown that NEAT1 is closely associated with chemoresistance in a variety of cancers, and can regulate the sensitivity of cancers such as myeloma and glioblastoma to chemotherapeutic agents through multiple pathways and pleiotropic effects, thus affecting the efficacy of cancer chemotherapy [28–30]. Therefore, in-depth study of the specific molecular regulation mechanism of NEAT1 in cancer chemotherapy resistance is of great value for screening effective and potential new targets for cancer chemotherapy sensitization, and provides new ideas and new directions for cancer chemotherapy.

Based on the main molecular mechanism of cancer chemotherapy resistance, this paper systematically expounds the molecular regulation and function of lncRNA NEAT1 in cancer chemotherapy resistance, which provides a scientific basis for the development of NEAT1 as a new target for cancer chemotherapy sensitization, and provides new ideas and insights for solving the problem of cancer chemotherapy resistance.

## The biogenesis and function of NEAT1

**Fig. 2:**
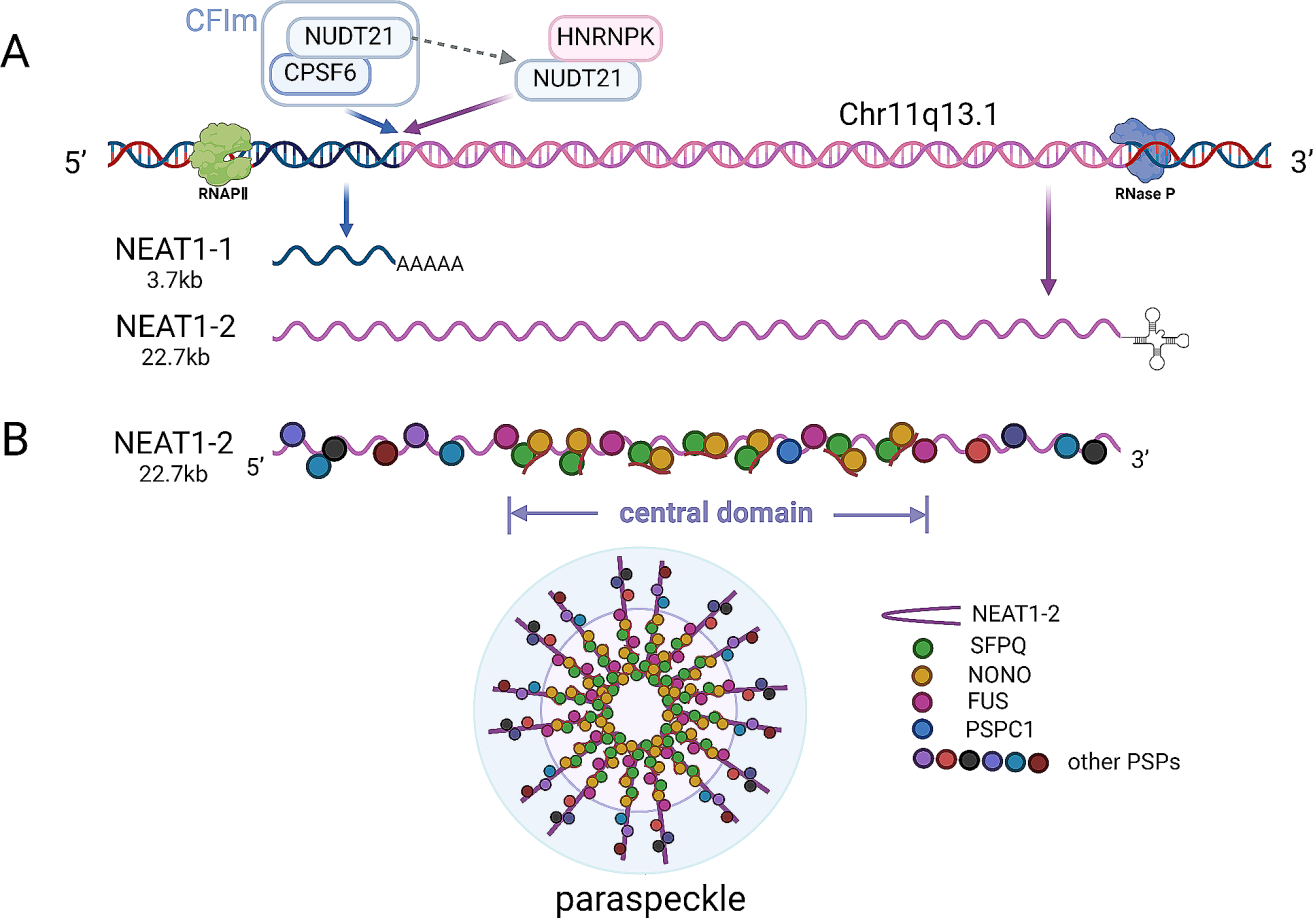
Biogenesis of NEAT1. (**A**) Location of NEAT1-1 and NEAT1-2 at the MEN site. (**B**) paraspeckle proteins(PSP) participates in the synthesis of a single NEAT1-2 ribonucleoprotein complex and a paraspeckle model

NEAT1 is located in the 11q13.1 region of human chromosome and interacts with RNA binding proteins to form important structural scaffolds of para-spots [31]. NEAT1 has two transcript variants with the same transcription start site, namely the short variant NEAT1-1 (MENε, 3.7 kb) and the long variant NEAT1-2 (MENβ, 22.7 kb) [32]. NEAT1-2 is the basic structural component of the paraspot, and the middle domain contains multiple sites that can bind to the paraspot proteins SFPQ, NONO, PSPC1 and FUS. These binding sites are necessary and sufficient for the formation, stability and integrity of the paraspot (Fig. 2) [33].

**Fig. 3:**
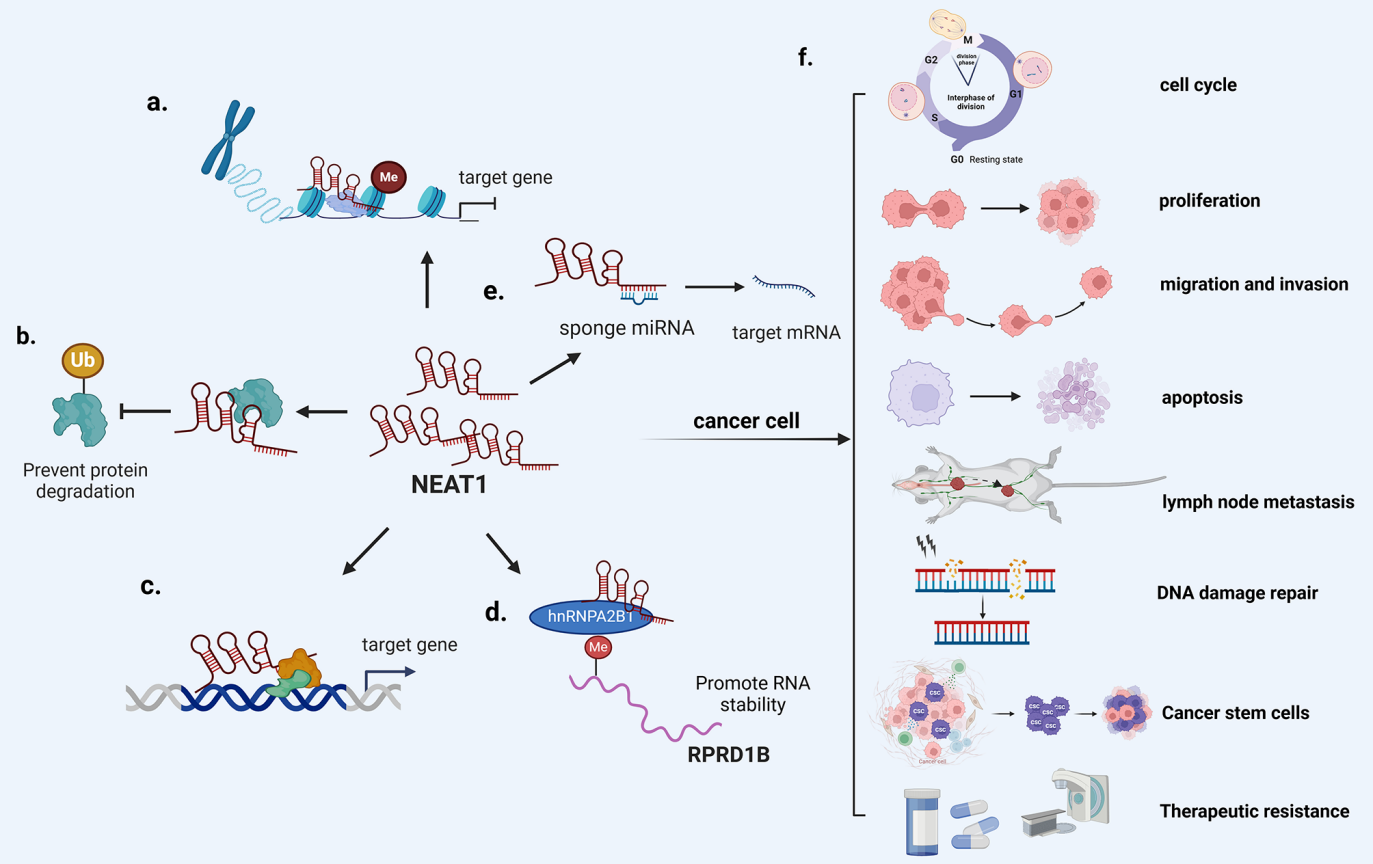
Mechanisms of NEAT1 in cancer. (**a**) Mediating epigenetic modification. (**b**) Regulating protein activity. (**c**) Regulate target gene transcription. (**d**) Regulate the stability of mRNA. (**e**) Sponge miRNA. (**f**) NEAT1 participates in the biological processes of cancer

At present, NEAT1 has been shown to be able to regulate biological functions of cancer cells by interacting with mRNA, DNA and proteins (Figure 3). For example (1) Regulate target gene transcription. In melanoma, NEAT1 binds to BRD4 through the BET domain, blocking WDR5 in a non active form, thereby mediating inhibitory effects on downstream target genes of BRD4 [34]; (2) Mediating epigenetic modification. NEAT1 mediates the trimethylation of histone H3 lysine 27 in the promoter region of target-specific genes Axin2 and GSK3B by acting as a scaffold and recruiting chromosome modification enzyme EZH2 to promote the nuclear transport of β-catenin, thereby promoting the growth and invasion of glioblastoma [35]. Similarly, NEAT1 directly interacts with BRG1 and significantly inhibits the expression of GADD45A by enhancing local H3K27me3 modification and reducing H3K4me3 modification, ultimately promoting the proliferation and inducing apoptosis of gastric cancer cells [36]; (3) Regulating protein activity. The hairpin A of NEAT1 can interact with the M1 domain of PGK1, prevent the degradation of PGK1 protein by inhibiting ubiquitination, and promote the expression of PGK1 protein to enhance the proliferation and glycolysis of glioma cells [37]; (4) Regulate the stability of mRNA. NEAT1 can recruit hnRNPA2B1 to bind to RPRD1B to promote the stability of RPRD1B mRNA, thereby promoting the metabolism of fatty acids and lymph node metastasis in gastric cancer [38]; (5) Sponge miRNA. NEAT1 acts as a competitive endogenous RNA (ceRNA) to regulate the transcription of target genes [39, 40]. NEAT1 competitively binds to miR-146b-5p to attenuate the inhibitory effect of miR-146b-5p on TRAF6, thereby increasing the expression of TRAF6 and promoting the proliferation, migration and invasion of pancreatic cancer cells [41]. In addition, NEAT1 can target miR-152-3p to up-regulate the expression of CDK19 and promote the cell viability, cycle and apoptosis of ovarian cancer cells, which can be reversed by evodiamine [42]. Therefore, the regulation of NEAT1 on cancer chemotherapy resistance may be achieved through these pathways.

##  Expression and clinical characterization of NEAT1 in different cancers

With the development of genome-wide analysis technology, lncRNAs have been proposed as biomarkers for early detection and prognosis of various cancers [43, 44]. NEAT1 is dysregulated in a wide range of cancers and is significantly associated with complex clinicopathological features. A number of studies have shown that NEAT1 affects various clinicopathological features such as TMN stage, tumor size, lymph node metastasis, and distant metastasis in cancer patients. Most cancer patients with high NEAT1 expression often indicate poor prognosis and shorter survival (Table 1). Therefore, the dysregulation of NEAT1 can be regarded as a robust and credible adverse prognostic factor in human cancer, which will help to develop effective strategies for the diagnosis, treatment and prognosis of cancer in the future.

**Table 1:** Clinical characteristics of NEAT1 in diverse cancer

Cancer type	Expression	Numbers of clinical samples	Clinical characteristics			Ref.
			clinical stage	metastasis	survival	
Prostate cancer	upregulation	130 paired tissue specimens of PC patients and matched normal prostate tissue	TNM III-IV↑	lymph node metastasis, distant metastasis	overall survival↓	[45]
Colorectal cancer	upregulation	serum samples from 406 CRC patients and healthy subjects	TNM III-IV↑	distant metastasis	overall survival↓	[46]
	upregulation	tissue and serum samples from 271 CRC patients and healthy subjects	tumor extent↑	distant metastasis	NA	[47]
Breast cancer	upregulation	serum samples from 178 patients with BC, benign breast diseases and healthy subjects	TNM II-IV↑	NA	NA	[48]
Oral squamous cell carcinoma	downregulation	34 pairs of OSCC tissues and adjacent non cancerous matching tissues samples	NA	NA	overall survival↓	[49]
Nasopharyngeal carcinoma	upregulation	128 specimens of NPC tissue and nasopharyngeal epithelial tissue	TNM III-IV↑	distant metastasis	overall survival↓	[50]
Multiple myeloma	upregulation	bone marrow samples from 144 MM patients and healthy subjects	ISS stage↑	NA	overall survival↓	[51]
	upregulation	bone marrow and blood samples from 81 MM patients and healthy subjects	ISS stage↑	NA	progression-free survival ↓	[28]
Laryngeal squamous cell carcinoma	upregulation	tissue samples from 102 LSCC patients	TNM III-IV↑	lymph node metastasis	overall survival↓	[52]

##  The molecular mechanism of NEAT1 in human cancer chemotherapy resistance

**Fig. 4:**
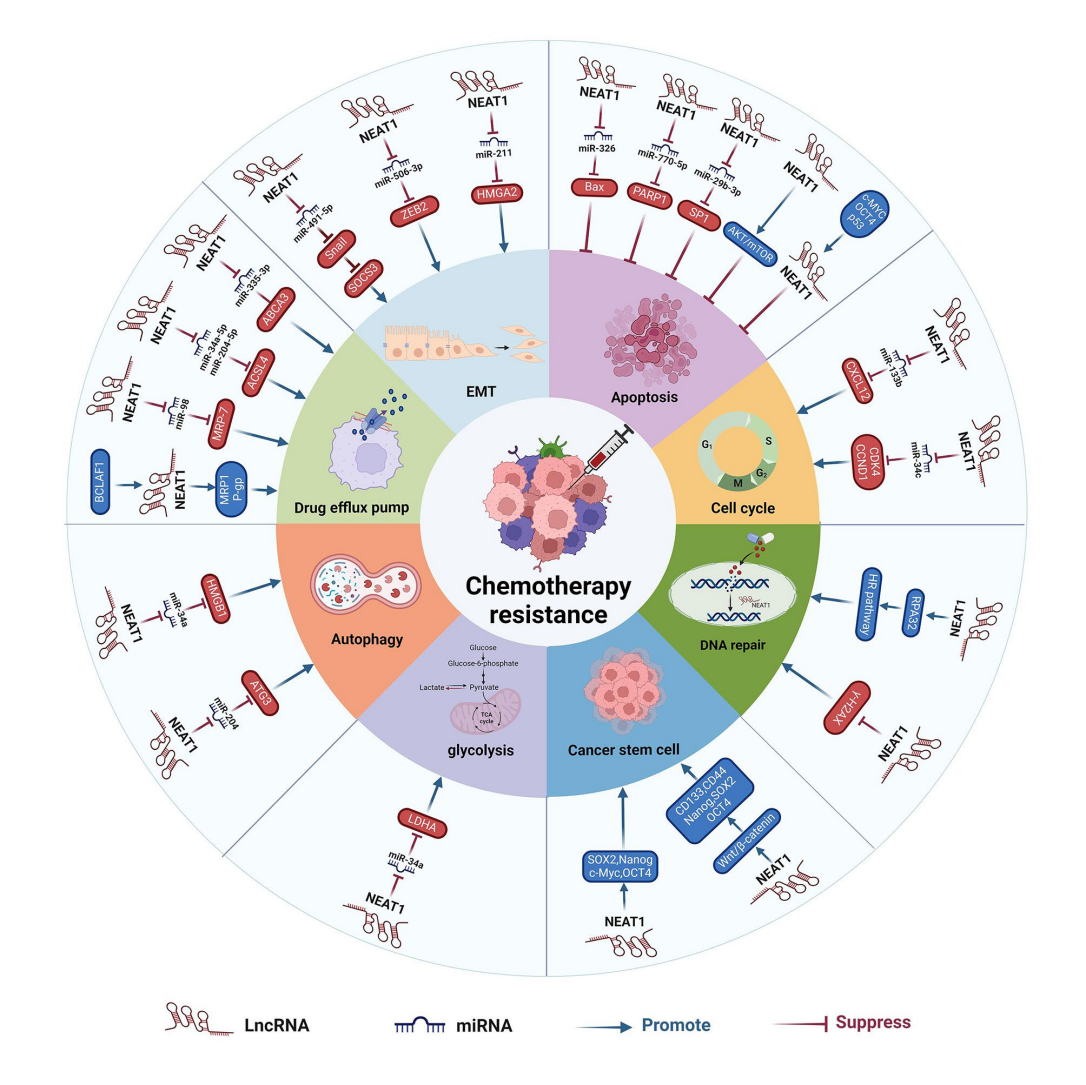
The mechanism of NEAT1 mediated cancer chemotherapy resistance

The role of NEAT1 in chemotherapy resistance of human cancer involves a variety of mechanisms, including inhibition of apoptotic pathways, increased ATP-driven drug efflux, enhanced DNA damage repair, and induction of autophagy (Table 2; Fig. 4).

**Table 2:** The mechanism of NEAT1 mediated chemotherapy resistance

Cancer type	Related drugs	Expression	Effect	Related genes and pathways	Ref.
Non-small cell lung cancer	paclitaxel	upregulation	apoptosis	AKT/mTOR	[53]
	paclitaxel	upregulation	apoptosis	AKT	[54]
	cisplatin	upregulation	cellular stemness	β-catenin, GSK3β, CD133, CD44, SOX2, Nanog, OCT4	[55]
Breast cancer	paclitaxel	upregulation	cell cycle	miR-133b, CXCL12	[56]
	5-Fu	upregulation	EMT	miR-211, HMGA2	[57]
Bladder cancer	cisplatin	upregulation	apoptosis	c-MYC, OCT4, p53	[58]
Ovarian cancer	cisplatin	upregulation	apoptosis	miR-770-5p, PAPR1	[59]
Neuroblastoma	cisplatin	upregulation	apoptosis	miR-326	[60]
Hepatocellular carcinoma	sorafenib	upregulation	autophagy	miR-204, ATG3	[61]
	5-Fu	upregulation	drug transport	P-gp, MRP1	[62]
Lymphoblastic leukemia	multidrug resistance	upregulation	drug transport	miR-335-3p, ABCA3	[63]
Colorectal cancer	5-Fu	upregulation	autophagy	miR-34a, HMGB1	[64]
	5-Fu	upregulation	cellular stemness	ALDH1, c-Myc, H3K27ac, SOX2, Nanog, OCT4	[65]
Osteosarcoma	cisplatin	upregulation	cell cycle	miR-34c, CCND1, CDK4	[66]
	bleomycin	upregulation	DNA repair	γ-H2A.X	[67]
Prostatic cancer	docetaxel	upregulation	drug transport	miR-34a-5p, miR-204-5p, ACSL4, MRP4, BCRP	[68]
Endometrial cancer	paclitaxel	upregulation	drug transport	miR-98, MRP-7, PLAUR	[69]
Multiple myeloma	bortezomib	upregulation	apoptosis	miR-29b-3p, SP1	[70]
	Bortezomib carfilzomib	upregulation	DNA repair	RPA32	[71]
Pancreatic cancer	gemcitabine	upregulation	EMT	miR-491-5p, Snail, SOCS3	[72]
	gemcitabine	upregulation	EMT	miR-506-3p, ZEB2	[73]
Cervical cancer	5-Fu	upregulation	glycolysis	miR-34a, LDHA	[74]

### NEAT1 inhibits cell apoptosis

Apoptosis is a gene-controlled, autonomous and orderly death process that is used to maintain the stability of the internal environment, and the escape of cancer cells from apoptosis is considered to be one of the hallmarks of cancer [75]. During chemotherapy, the DNA of cancer cells will be subjected to irreparable chemical damage, thereby inducing apoptosis. However, cancer cells will take a variety of ways to escape apoptosis, including inhibiting pro-apoptotic signals, inducing anti-apoptotic signals, or affecting apoptotic signal transduction pathways through gene mutations. The combined effects of these mechanisms promote the development of chemotherapy resistance in cancer cells [76, 77]. The apoptosis pathway mainly includes mitochondrial pathway and death receptor pathway. When the mitochondrial apoptotic pathway is activated, BAX is relocated to the mitochondrial outer membrane and forms an oligomeric complex with BAK1. The complex is inserted into the outer membrane pores of the mitochondria, causing changes in mitochondrial osmotic pressure and loss of transmembrane potential, prompting the release of cytochrome c from the mitochondria to the cytoplasm. Cytochrome c entering the cytoplasm binds to Apaf1 to form an apoptotic complex, which recruits and activates the precursor of cysteine aspartic protease-9 (caspase-9), thereby activating Caspase-3 and Caspase-7, triggering the Caspase cascade and ultimately leading to apoptosis [78]. NEAT1 can inhibit apoptosis and promote chemotherapy resistance of cancer by regulating the expression of key apoptosis-related factors in the mitochondrial apoptosis pathway. In non-small cell lung cancer (NSCLC), NEAT1 can inhibit apoptosis and induce paclitaxel resistance in NSCLC cells by inhibiting the expression of apoptosis-related proteins cleaved caspase3 and cleaved PARP and activating the AKT/mTOR signaling pathway [53, 54]. In ovarian cancer (OC) cells, NEAT1 up-regulates the expression of PARP1 and inhibits the expression of apoptosis-related proteins BAX and c-caspase3 by targeting miR-770-5p, thereby inhibiting apoptosis and inducing cisplatin resistance in OC cells [59]. Yang et al. [60] found that high expression of NEAT1 can inhibit the apoptosis of neuroblastoma (NB) cells and promote the resistance of NB cells to cisplatin by targeting down-regulating the expression level of miR-326 and inhibiting the expression of pro-apoptotic protein Bax. Multiple myeloma (MM) is a malignant tumor with abnormal proliferation of plasma cells in bone marrow. It has been found that high expression of NEAT1 in MM serum samples and cells can promote the chemoresistance of MM cells. The specific mechanism is that NEAT1 can up-regulate the expression of SP1 and down-regulate the expression of apoptosis-related proteins c-PARP and c-caspase3 through sponge miR-29b-3p, thereby inhibiting the apoptosis of MM cells and enhancing the tolerance of MM cells to bortezomib [70]. The cisplatin resistance of bladder cancer (BC) cells is also related to the regulation of NEAT1 on apoptosis escape. In cisplatin-resistant BC cells, up-regulated oncogene transcription factors c-MYC, OCT4 and p53 can be enriched in the NEAT1 promoter region and activate its transcription, inhibit apoptosis, and promote cisplatin resistance in BC cells [58].

###  NEAT1 promotes cell cycle progression

Uncontrolled cell proliferation caused by cell cycle disorders is an important feature of cancer [79]. The abnormal changes of proteins, enzymes, cytokines and cell cycle signaling pathways involved in cell cycle regulation are closely related to the occurrence, development and chemotherapy resistance of cancer [80]. NEAT1 can induce chemotherapy resistance by promoting the cell cycle progression of cancer cells. The G0/G1 phase of the cell cycle is an important period for cell volume growth and preparation for DNA replication and synthesis. NEAT1 can affect the chemoresistance of cancer cells by regulating the GO/G1 phase of the cell cycle. NEAT1 derived from BC paclitaxel-resistant SKBR-3/PR cell exosomes can induce paclitaxel resistance by regulating the GO/G1 phase of the cell cycle.CXCL12 plays an important regulatory role in promoting cancer chemotherapy resistance. In BC paclitaxel-resistant SKBR-3/PR cells, knockdown of NEAT1 can up-regulate the expression of miR-133b and down-regulate the expression of downstream CXCL12 protein, which promotes cell cycle arrest in G0/G1 phase and promotes the sensitivity of SKBR-3/PR cells to paclitaxel [56]. NEAT1 in osteosarcoma (OS) cells can also enhance the resistance of OS cells to cisplatin by promoting the G0/G1 phase of the cell cycle [66].

###  NEAT1 affects drug efflux

The reduction of intracellular accumulation of chemotherapeutic drugs is one of the main causes of cancer drug resistance, which is closely related to the drug efflux system [81]. Adenosine triphosphate binding cassette (ABC) transporters in the drug efflux system can regulate the absorption, distribution and clearance of drugs. Its family members, multidrug resistance protein 1 (ABCB1/MDR1/P-gp) and multidrug resistance-associated protein 1 (ABCC1/MRP1), can transport chemotherapeutic drugs with different structures and functions from intracellular to extracellular by using the energy hydrolyzed by ATP, significantly reducing intracellular drug concentration and playing an important role in the development of cancer chemoresistance [82, 83]. NEAT1 can promote the efflux of chemotherapeutic drugs by regulating the expression and activity of ABC transporters in cancer cells, thereby causing cancer chemotherapy resistance. MRP subfamily members of ABC transporters are expressed in a variety of cancers as major players in cancer multidrug resistance. Huang et al. [69]. found that multidrug resistance protein 7 (MRP7), which is highly expressed in paclitaxel-resistant endometrial cancer (EC) cells, can promote the paclitaxel resistance of EC cells, while NEAT1 can up-regulate the expression level of MRP7 through sponge miR-98, thereby further enhancing the paclitaxel resistance of EC cells. ACSL4 has been shown to enhance chemoresistance of cancer cells by affecting the expression of ABC transporters [84]. The expression of NEAT1 is significantly up-regulated in prostate cancer (PCa) tissues and cells, and promotes the resistance of PCa cells to docetaxel. The specific mechanism is that NEAT1, as a ceRNA, up-regulates the expression of ACSL4 by sponge miR-34a-5p or miR-204-5p, and promotes the high expression of downstream ABC transporters MRP4 and BCRP, thereby enhancing the resistance of PCa cells to docetaxel [68]. BCLAF is a multifunctional BCL2-related transcription factor that is up-regulated in hepatocellular carcinoma (HCC). It enhances the transcription of NEAT1 by targeted binding to the promoter region of NEAT1, and further promotes the expression levels of MRP1 and P-gp to induce 5-Fu resistance in HCC cells [62]. ABCA3 is a member of the ABC1 subfamily and is up-regulated in the multidrug-resistant group of childhood acute lymphoblastic leukemia. It is a new tumor prognostic marker. NEAT1 can target up-regulate the expression of ABCA3 through sponge miR-335-3p, and ultimately lead to chemotherapy resistance in children with acute lymphoblastic leukemia [63].

###  NEAT1 promotes DNA damage repair

The mechanism of DNA repair is triggered after DNA damage, and failure to repair DNA properly is a major cause of genetic mutations and oncogene activation. Chemotherapy drugs usually work by inducing DNA damage in cancer cells, and cancer cells can reduce the effect of chemotherapy by enhancing DNA damage repair [85]. DNA damage is mainly repaired by five pathways: homologous recombination (HR), nucleotide excision repair (NER), non-homologous end joining (NHEJ), base excision repair (BER) and mismatch repair (MMR) [86]. NEAT1 promotes chemotherapeutic resistance in cancer mainly by enhancing the homologous recombination ability of cancer cells. In MM cells, NEAT1 enhances the DNA damage repair process by upregulating the expression of RPA32, a key molecule in the HR pathway, thus promoting cell resistance to bortezomib and carfezomib. However, silencing NEAT1 can reduce the expression of RPA32, induce a large amount of DNA damage, and enhance the sensitivity of MM cells to bortezomib and carfilzomib [71]. Recent studies have shown that NEAT1 is positively correlated with the HR-related proteins RAD51 and FOXM1 in OC, and that knockdown of NEAT1 sensitizes cancer cells to platinum-based chemotherapeutic agents by inhibiting the HR process [87]. In addition, NEAT1 can also reduce the number of DNA damage lesions by promoting the expression of HRR related protein ATR, thereby promoting the resistance of OS cells to bleomycin [67].

###  NEAT1 enhances EMT

EMT gives cancer cells the ability to invade and metastasize, which is an important factor in promoting cancer progression and causing chemotherapy failure [88]. During chemotherapy, cancer cells that have undergone EMT gain the ability to resist apoptosis, and this resistance often leads to increased tolerance of cancer cells to chemotherapy. NEAT1 can promote chemotherapy resistance of cancer cells by regulating the EMT process. SOCS3 is a cytokine-induced protein that can inhibit the development of cancer by regulating cell proliferation and drug resistance [89]. In pancreatic cancer (PC) cells, NEAT1 can enhance the resistance of PC cells to gemcitabine by promoting EMT process. The specific mechanism is that NEAT1 inhibits downstream SOCS3 expression through sponge miR-491-5p targeting. Thus, the expression of EMT-related proteins E-cadherin is down-regulated, Snail, vimentin and fibronectin are up-regulated, and the resistance of PC cells to gemcitabine is enhanced [72]. Similarly, NEAT1 can also up-regulate the expression of transcription regulator ZEB2 in PC cells by targeting sponge miR-506-3p, to promote the EMT process of cells and induce cell resistance to gemcitabine [73]. In BC, high expression of NEAT1 is associated with invasive breast cancer phenotype and poor patient prognosis. HMGA2 is one of the members of the high mobility group protein superfamily with the HMG domain. It is a non-histone protein that can bind DNA and modify chromatin conformation to regulate gene expression. HMGA2 is closely related to tumor formation, and abnormally expressions in various cancer tissues, which can promote the invasion and migration of BC cells [90]. Down-regulation of NEAT1 inhibited the EMT process and increased the sensitivity of BC cells to 5-Fu by targeting the miR-211/HMGA2 axis [57].

###  NEAT1 induces autophagy

Autophagy, also known as type II programmed cell death, degrades damaged proteins and dysfunctional organelles through autophagolysosomes, providing cells with energy and nutrition to maintain cell homeostasis [91]. Autophagy is a double-edged sword in cancer, in the early stage of cancer development, Autophagy can eliminate damaged cells to play a role in cancer suppression. However, in the later stages of cancer progression, autophagy acts as a cancer protective factor. Under the stimulation of chemotherapy drugs, cancer cells accelerate drug decomposition by increasing their autophagy level, thus improving the tolerance of cancer cells to chemotherapy drugs [92, 93]. NEAT1 can enhance chemotherapy resistance of cancer cells by inducing autophagy. In colorectal cancer (CRC) tissues and cells, high expression of NEAT1 promotes resistance to 5-Fu in CRC cells by inducing autophagy. HMGB1 is a member of the high mobility group protein superfamily, which has been shown to promote the resistance of cancer to radiotherapy and chemotherapy [94]. Knockdown of NEAT1 can target down-regulate the expression of miR-34a and up-regulate the expression of HMGB1, thereby inhibiting the expression of autophagy related proteins Bclin-1 and ULK1 and the ratio of LC3II/I, and enhancing the sensitivity of CRC cells to 5-Fu [64]. NEAT1 can also promote the expression of downstream ATG3 through sponge miR-204, induce autophagy in HCC cells, and promote the resistance of HCC cells to sorafenib [61].

###  NEAT1 enhances cancer stem cell properties

Cancer stem cells (CSCs) are a group of cancer cells with the ability of self-renewal and differentiation, which are closely related to cancer metastasis, recurrence and chemotherapy resistance [95, 96]. After chemotherapy, CSCs in cancer tissues protect themselves from the destructive effects of chemotherapeutic drugs and enhance their resistance to chemotherapeutic drugs through many ways like scavenging reactive oxygen species, inducing cell dormancy and activating developmental signaling pathways [97]. Cancer stemness transcription factors such as SOX2, OCT4, and Nanog serve as markers for CSCs, which can maintain the multipotency and self-renewal of CSCs through various pathways such as promoting the EMT process, modulating the signaling pathway, and regulating the cell cycle [98]. NEAT1 can induce chemoresistance in cancer cells by regulating the expression of these stemness factors. In CRC tissues and cells, high expression of NEAT1 is associated with short recurrence-free survival in CRC patients. NEAT1 is able to act as a scaffold to affect chromatin remodeling in cancer cells, increase the expression of H3K27ac and lead to increased levels of acetylation in the ALDH1 and c-Myc promoter regions, which in turn upregulates the expression of the cancer stemness factors SOX2, Nanog, and OCT4, and enhances the stemness of colorectal cancer cells and increases their resistance to 5-Fu [65]. NEAT1 can also affect CSCs by activating the signaling pathway to induce chemoresistance of cancer cells. The specific mechanism is that NEAT1 promotes the sphere-forming ability of A549/CDDP cells by activating the Wnt/β-catenin signaling pathway, increases the expression levels of CSCs-related stemness factors CD133, CD44, SOX2, Nanog, OCT4, induces the stemness of A549/CDDP cells, and further enhances its resistance to cisplatin [55].

###  NEAT1 promotes aerobic glycolysis

Cancer cells have different metabolic patterns from normal cells. Even under aerobic conditions, rapidly proliferating cancer cells tend to consume glucose to produce lactic acid, which is called Warburg effect or aerobic glycolysis [99]. A key mechanism of chemotherapeutic resistance is the metabolic reprogramming of cancer, which involves changes in cellular metabolic pathways, including increased aerobic glycolysis, to provide cancer cells with the necessary energy to escape the effects of chemotherapy [100, 101]. NEAT1 can affect the chemotherapy resistance of cancer by promoting aerobic glycolysis. LDHA as a key enzyme in the glycolysis process, plays an important role in promoting the growth of cancer cells [102]. In cervical cancer (CCA), NEAT1 targets and upregulates LDHA expression via sponge miR-34a, which promotes the cellular glycolysis rate and enhances 5-Fu resistance in CCA cells [74].

##  Conclusion and future perspectives

As an important means of cancer treatment, chemotherapy has a significant effect on improving the quality of life and prognosis of patients. However, the emergence of chemotherapy resistance is an important cause of clinical chemotherapy failure. At present, lncRNA has been shown to play an important regulatory role in a variety of life activities, especially in the regulation of chemotherapy resistance of cancer cells. LncRNA can regulate the chemotherapy resistance of cancer cells through a variety of ways, and can be used as a potential sensitization target to improve the efficacy of chemotherapy. NEAT1 is one of the most widely studied lncRNAs, and it is also the most up-regulated lncRNA in the pan-cancer genome data set, which plays an important regulatory role in tumorigenesis and development. Studies have shown that NEAT1 is closely related to the malignant biological behavior of many cancers and the poor prognosis of patients, and is a potential cancer therapeutic target. In this review, we explore the different mechanisms by which NEAT1 induces drug resistance in a variety of tumors. The results showed that NEAT1 could cause resistance to a variety of chemotherapeutic agents, such as paclitaxel, cisplatin, bortezomib, 5-FU, bleomycin, and so on. NEAT1 can regulate the resistance of cancer cells to chemotherapeutic drugs by inhibiting apoptosis, inducing autophagy, promoting EMT, enhancing the characteristics of cancer stem cells, and promoting drug efflux. Therefore, NEAT1 may be a new potential therapeutic target for overcoming cancer chemotherapy resistance. Targeting NEAT1 can not only effectively anti-tumor, but also enhance the effect of chemotherapy, thus contributing to the comprehensive treatment of cancer and improving the prognosis of patients.

At present, new therapies targeting lncRNAs are a research hotspot in cancer treatment. Therapeutic strategies for functional lncRNA development, such as antisense oligonucleotides (antagomiRs/ASO), RNA nanotechnology, and CRISPR-Cas9 technology, have achieved certain results. Li et al. [103]. found that inhibition of NEAT1 expression by CRISPR-Cas9 technology can make anti-radiation cancer cells more sensitive to radiation, and reduce their cell proliferation and stem cell marker expression, indicating that CRISPR-Cas9 gene editing technology can be used to interfere with dysfunctional NEAT1 in cancer. The study of NEAT1-related ceRNA network can also open up new avenues for cancer treatment and overcoming drug resistance. NEAT1 competes with specific miRNAs. These competitive endogenous RNA networks form a complex and highly regulated mechanism to control gene expression and cell function, which provides new possibilities for targeting NEAT1 in combination with traditional chemotherapy to overcome cancer chemotherapy resistance.In addition, NEAT1 is secreted in almost all biological fluids, which allows it to be used as a biomarker for clinical diagnosis of cancer and development of chemotherapy resistance in cancer, and its expression level can differentiate between chemo-sensitive and chemo-resistant tumors, which can help in the early detection of chemo-resistance and timely change of therapeutic strategies.Some of the current studies have accurately identified inhibitors targeting NEAT1 and found their value in reversing tumor chemotherapy resistance, providing an important theoretical basis for clinical development of new anticancer drugs.

In recent years, many advances have been made in the study of the mechanism of action of NEAT1 in cancer development, but the study of the mechanism of action of NEAT1 in cancer chemotherapy resistance is still in its infancy. There are still a number of challenges that need to be addressed.First, the molecular mechanism of NEAT1 in cancer chemoresistance is a complex regulatory network, and although a large number of NEAT1-related signaling pathways have been found to be associated with cancer chemoresistance, there are still many unknown signaling pathways and potential molecular targets waiting to be further explored; Second, NEAT1 has the potential to become a biomarker and sensitization target for clinical cancer diagnosis and treatment, but more studies are needed to further validate whether its reliability and sensitivity are sufficient for clinical applications; Finally, although the currently discovered NEAT1-specific inhibitors have shown significant anti-cancer effects, due to the lack of basic research on the potential downstream pathways of NEAT1, there is a certain risk in gene therapy targeting NEAT1 compared with traditional drugs. Due to the lack of clinical data, the efficacy and safety of drugs targeting NEAT1 in humans is unknown, and unexpected side effects may occur when applied in the clinic. In addition, specific targeting methods and delivery systems need to be further improved to ensure that only NEAT1 is affected.

In summary, in-depth understanding and investigation of the specific role of NEAT1 in chemotherapy resistance in different cancers can help to reveal the intrinsic mechanism of cancer chemotherapy resistance, which is of great significance to the clinical research and development of targeted chemotherapy sensitizing drugs, and to improve the efficacy of chemotherapy and prognosis of cancer patients. This article describes the molecular mechanism of NEAT1 in cancer chemoresistance, and explores the possibility of NEAT1 as a target for cancer chemotherapy sensitization, in order to provide a theoretical basis from the perspective of lncRNA, find new targets for chemotherapy sensitization, and improve clinical chemotherapy efficacy.

## Data Availability

No datasets were generated or analysed during the current study.

## Abbreviations

Axin2: axis Inhibitor 2
Apaf1: apoptotic protease activating factor-1
ATG3: autophagy-related protein 3
ALDH1: aldehyde dehydrogenase 1
ACSL4: long-chain acyl-CoA synthetase 4
BRD4: bromodomain-containing protein 4
BRG1: brahma-related gene-1
BAX: bcl-2 assaciated X
BAK1: bcl-2 antagonist 1
BCLAF1: b-cell lymphoma 2-associated transcription factor 1
c-MYC: cellular-myelocytomatosis viral oncogene
CXCL12: stromal cell-derived factor-1
CDK19: cyclin dependent kinase 19
EMT: epithelial mesenchymal transition
EZH2: enhancer of zeste homolog
FUS: fused in sarcoma/translocated in liposarcoma
FOXM1: forkhead box protein M1
GADD45A: growth arrest and DNA damage inducible alpha
GSK3B: glycogen synthase kinase-3β
HMGA2: high mobility group protein A2
HMGB1: high mobility group box 1
hnRNPA2B1: heterogeneous nuclear ribonucleoprotein A2B1
LDHA: lactate dehydrogenase A
NONO: non-POU domain-containing octamer-binding protein
OCT4: octamer-binding transcription factor 4
PSPC1: paraspeckle component 1
PGK1: phosphoglycerate kinase 1
PARP1: poly ADP-ribose polymerase-1
P53: tumor protein p53
RPRD1B: regulation of nuclear pre-mRNA domain containing 1B
RAD51: RAD51 recombinase
SP1: transcription specificity protein 1
SOCS3: suppressor of cytokine signaling 3
SOX2: sex determining region Y-box 2
TRAF6: TNF receptor associated factor 6
WDR5: WD repeat domain 5
ZEB2: zinc finger E-box binding homeobox 2
